# Treatment of diabetes mellitus-induced erectile dysfunction using endothelial progenitor cells genetically modified with human telomerase reverse transcriptase

**DOI:** 10.18632/oncotarget.9909

**Published:** 2016-06-07

**Authors:** Yan Zhang, Zhi Chen, Tao Wang, Jun Yang, Rui Li, Shaogang Wang, Jihong Liu, Zhangqun Ye

**Affiliations:** ^1^ Institute of Urology, Tongji Hospital, Tongji Medical College, Huazhong University of Science and Technology, Wuhan, China; ^2^ Department of Urology, Tongji Hospital, Tongji Medical College, Huazhong University of Science and Technology, Wuhan, China; ^3^ Department of Gerontology, Tongji Hospital, Tongji Medical College, Huazhong University of Science and Technology, Wuhan, HuBei, China

**Keywords:** diabetes mellitus, erectile dysfunction, endothelial progenitor cells, human telomerase reverse transcriptase, paracrine, Pathology Section

## Abstract

The efficacy of treatments for diabetes mellitus-induced erectile dysfunction (DMED) is quite poor, and stem cell therapy is emerging as a useful method. In this study, we used endothelial progenitor cells (EPCs) overexpressing human telomerase reverse transcriptase (hTERT) for the treatment of DMED. Rat EPCs were transfected with hTERT (EPCs-hTERT). EPCs-hTERT secreted more growth factors and demonstrated enhanced proliferation and resistance to oxidative stress. Twenty-four male DMED rats were subjected to four treatments: DMED (DMED group), EPCs (EPCs group), EPCs transduced with control lentivirus (EPC-control group) and EPCs-hTERT (EPCs-hTERT group). A group of healthy rats were used as the normal control group. The erectile function in the EPCs-hTERT group was markedly increased compared with the EPCs and EPCs-control groups. The EPCs-hTERT group exhibited more growth factors, smooth muscle content and retained stem cells in penile tissues. The degree of apoptosis and collagen/smooth muscle ratio in penile tissues of the EPCs-hTERT group was considerably reduced. Endothelial nitric oxide synthase (eNOS) expression increased significantly in the EPCs-hTERT group. Taken together, these data showed that the enhanced paracrine effect, resistance to oxidative stress and proliferation of EPCs-hTERT may contribute to the improvements of erectile function in DMED rats.

## INTRODUCTION

Diabetes mellitus (DM) is a chronic disease that is associated with one of the most significant urological complications, erectile dysfunction (ED). Numerous studies reported that the prevalence of ED in diabetic men varies between 30 and 80%, which is increased six-fold compared with men without diabetes [1, 2]. The pathogenesis of diabetes mellitus-induced erectile dysfunction (DMED) is complex, and it is associated with the formation of advanced glycation end products, endothelial dysfunction, hypogonadism, peripheral neurological changes and decreased nitric oxide production [2, 3]. Phosphodiesterase type 5 inhibitors are used as a first-line treatment for ED, but these drugs are less effective in this population [4]. Therefore, it is necessary to explore new therapeutic targets for diabetic ED.

Stem cell therapy is one of the promising strategies with a demonstrated benefit in the treatment of DMED. Several studies reported that stem cells improved erectile function after intracavernous application [5, 6]. One of the possible mechanisms of stem cell therapy for ED is that stem cells incorporate into penile tissues and differentiate into smooth muscle or endothelial cells [7, 8]. Stem cells also secrete a variety of growth factors that exert protective effects *in vivo*, including vascular endothelial growth factor (VEGF), angiopoietin-1 (Ang-1), basic fibroblast growth factor (bFGF), brain-derived neurotrophic factor (BDNF), glial cell-derived neurotrophic factor (GDNF), and stromal derived factor-1 (SDF-1) [9].

EPCs are bone marrow-derived stem cells that migrate to sites of damaged endothelium and differentiate into vascular endothelial cells. EPCs play an important role in endothelial homeostasis and repair[10]. Several studies reported that the number of circulating EPCs in ED patients was significantly reduced compared with men without ED [11, 12], which suggests a role for these cells in ED progression. Increasing the number of EPCs may be a prospective treatment for ED. Gou *et al*. demonstrated that the transplantation of EPCs overexpressing VEGF165 restored erectile function in DMED rats via secretion of VEGF165 protein and enhanced neovascularization [13].

Human telomerase reverse transcriptase (hTERT) is the catalytic component of telomerase with reverse transcriptase activity. Stem cells overexpressing hTERT maintained proliferation capacity and the ability to differentiate into multiple cell lineages *in vivo* and *in vitro* without signs of malignant transformation [14, 15]. TERT plays a clear role in maintaining telomere length, and mounting evidence revealed that TERT exhibits telomeric-independent biological functions, such as enhancing survival, promoting proliferation, regulating glucose transport and decreasing intracellular reactive oxygen species (ROS) [16, 17].

Genetic modification strategies have emerged with the development of gene delivery methods and enhanced the efficacy of stem cells. Previous studies successfully applied genetically modified stem cells for ED treatment [13, 18, 19]. However, whether hTERT overexpression enhanced EPCs-based treatment of DMED was not demonstrated. The present study investigated the efficacy of the transplantation of EPCs overexpressing hTERT into penile tissues for the treatment of DMED.

## RESULTS

### Characterization of EPCs

**Figure 1:**
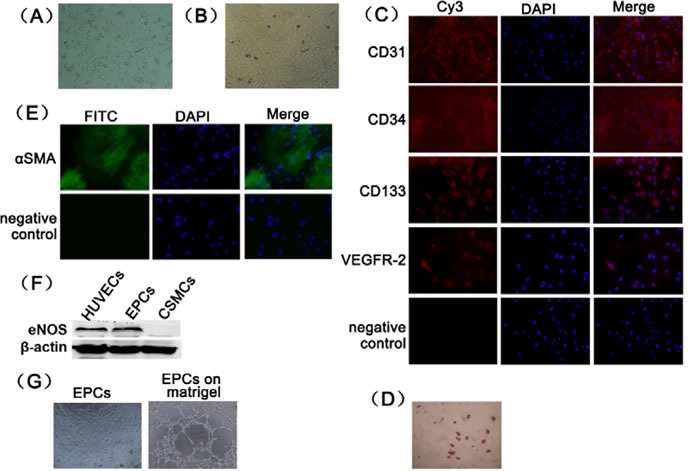
Primary culture and characterization of rat EPCs (A) Rat BM-MNCs from bone marrow after 7 days in culture (100×). (B) Rat BM-MNCs after 14 days of culture (100×). (C) CD31, CD34, CD133 and VEGFR-2 expression was detected using immunofluorescence (200×). (D) EPCs differentiation potential into adipocytes was demonstrated using oil red-O staining (100×). (E) EPCs differentiation potential into smooth muscle cells was demonstrated using immunofluorescence staining (200×). (F) The expression of eNOS in EPCs was identified using Western blotting analyses. Human umbilical vein endothelial cells was used as positive control. Cavernous smooth muscle cells was used as negative control. (G) Tube formation assay was examined microscopically (100×).

BM-MNCs exhibited a spindle appearance, and simultaneously colony-forming units became visible after 7 days in culture (Figure 1A). Some adherent cells grew into a cobblestone shape and exhibited a smooth cytoplasmic outline after 2 weeks (Figure 1B). These cells were called late EPCs, and we used late EPCs for further studies. EPCs expressed surface markers, such as CD31 (96.8±0.5%), CD34 (97.2±1.4%), CD133 (92.8±5.3%) and VEGFR-2 (91.5±3.7%) (Figure 1C), and this finding is consistent with previous studies [20, 21]. EPCs could differentiate into adipocytes (Figure 1D) and smooth muscle cells (Figure 1E). Endothelial nitric oxide synthase (eNOS) was also expressed in EPCs (Figure 1F). EPCs showed ability of forming capillary-like tubes (Figure 1G).

### Generation of genetically modified rat EPCs

**Figure 2:**
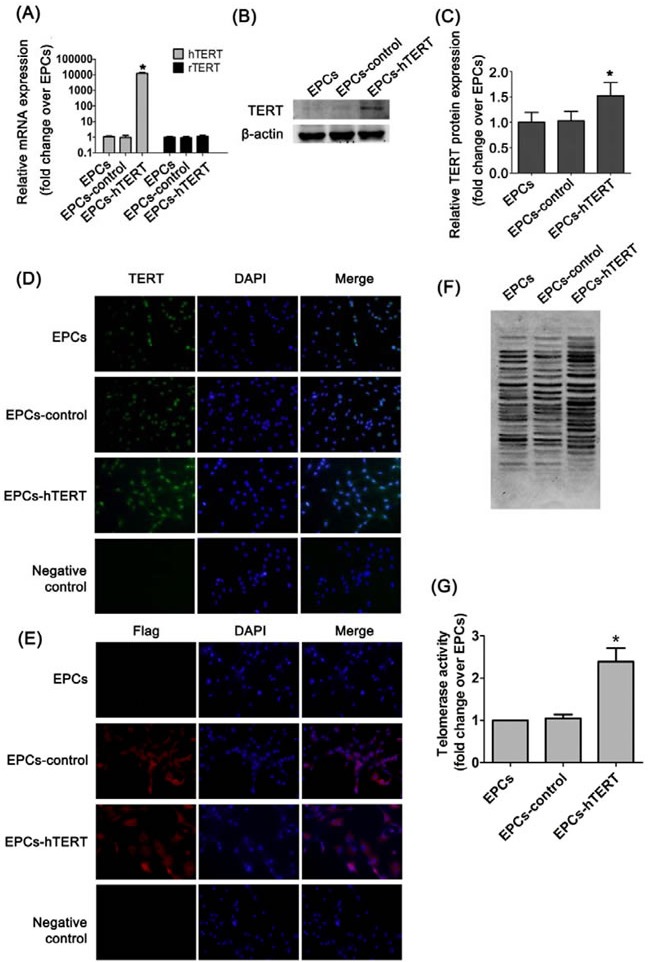
hTERT expression in EPCs after transduction (A) hTERT and rTERT mRNA expression were detected using real-time quantitative PCR analysis. (B) and (C) Western blotting analysis was used to evaluate TERT protein level in cells. (D) Immunofluorescence staining revealed the distribution of TERT protein in EPCs, EPCs-control and EPCs-hTERT (200×). (E) Immunofluorescence staining revealed the distribution of flag protein in EPCs, EPCs-control and EPCs-hTERT (200×). (F) and (G) Telomerase activity was detected using TRAP. *P<0.05 vs. EPCs.

EPCs stably expressing hTERT were created using a selectable marker (puromycin) after transduction with lentivirus. mRNA expression of hTERT in EPCs-hTERT was considerably increased compared with EPCs-control and EPCs (*P*<0.05). The Ct value of hTERT in EPCs and EPCs-control was greater than 35, and hTERT was not considered expressed in these two cells. mRNA expression of rTERT in EPCs-hTERT was not altered compared with EPCs-control and EPCs (Figure 2A). TERT is evolutionarily conserved in humans and rats, and the antibody against TERT binds to both hTERT and rTERT. The level of TERT protein expression in EPCs-hTERT was significantly increased compared with EPCs and EPCs-control (Figure 2B and 2C). Figure 2D demonstrates that TERT protein is primarily distributed in the nuclei of EPCs and EPCs-control, whereas EPCs-hTERT expressed TERT protein in the nucleus and cytoplasm. Flag was expressed in the cytoplasm of EPCs-control and EPCs-hTERT (Figure 2E). These results indicate that hTERT was expressed in the cytoplasm of EPCs-hTERT, whereas rTERT was expressed in the nucleus. rTERT expression was not altered after hTERT overexpression. Telomerase activity of EPC-hTERT was significantly increased compared with EPCs and EPCs-control (Figure 2F and 2G).

### Effects of hTERT overexpression on proliferation, endogenous ROS level, paracrine capacity and anti-oxidative stress

**Figure 3:**
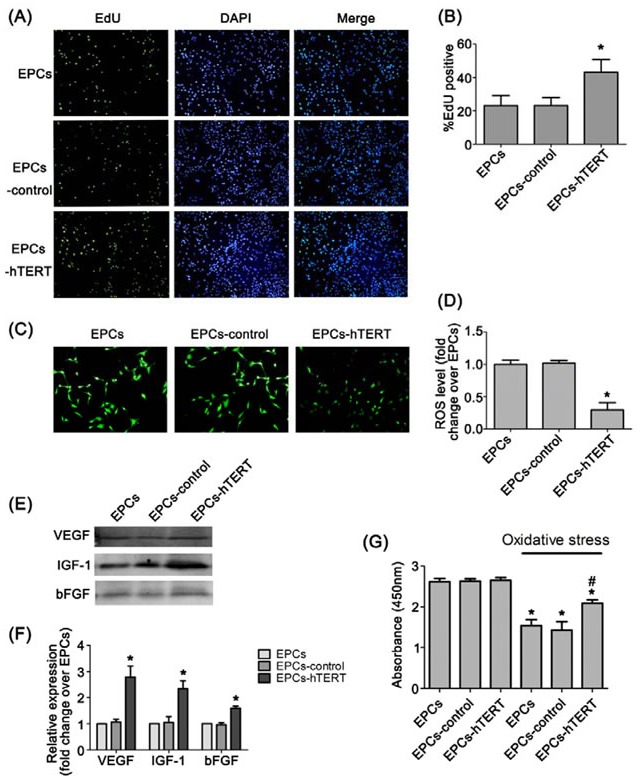
Characterization of hTERT-overexpressing rat EPCs (A) and (B) EdU staining indicates that hTERT overexpression increased EPCs proliferation (100×). (C) and (D) Intracellular ROS and fluorescence intensity analysis in EPCs, EPCs-control and EPCs-hTERT (100×). (E) and (F) The amount of VEGF, IGF-1 and bFGF secretion from EPCs-hTERT was detected using western blotting. (G) CCK-8 assay was used to evaluate the anti-oxidant capacity of EPCs-hTERT. *P<0.05 vs. EPCs, # P<0.05 vs. EPCs in oxidative stress.

The phenotypic profile and differentiation potential of EPCs was not altered after transduction with hTERT (Supplemental Figure 1). Figure 3A and 3B indicates that the percentage of EdU-positive cells in the S phase of the cell cycle was significantly increased in EPCs-hTERT compared with EPCs and EPCs-control (*P*<0.05). EPCs and EPCs-control revealed significant increases in intracellular ROS compared with EPCs-hTERT (*P*<0.05, Figure 3C and 3D). Western blotting revealed that EPCs-hTERT secreted more VEGF, insulin-like growth factor-1 (IGF-1) and bFGF than EPCs and EPCs-control (*P*<0.05, Figure 3E and 3F). We detected the survival of cells exposed to oxidative stress induced by hydrogen peroxide. CCK-8 assays demonstrated that EPCs-hTERT exhibited stronger anti-oxidant capacity than EPCs and EPCs-control (*P*<0.05, Figure 3G).

### Erectile function, histological changes and growth factors in penile tissues after transplantation

**Figure 4:**
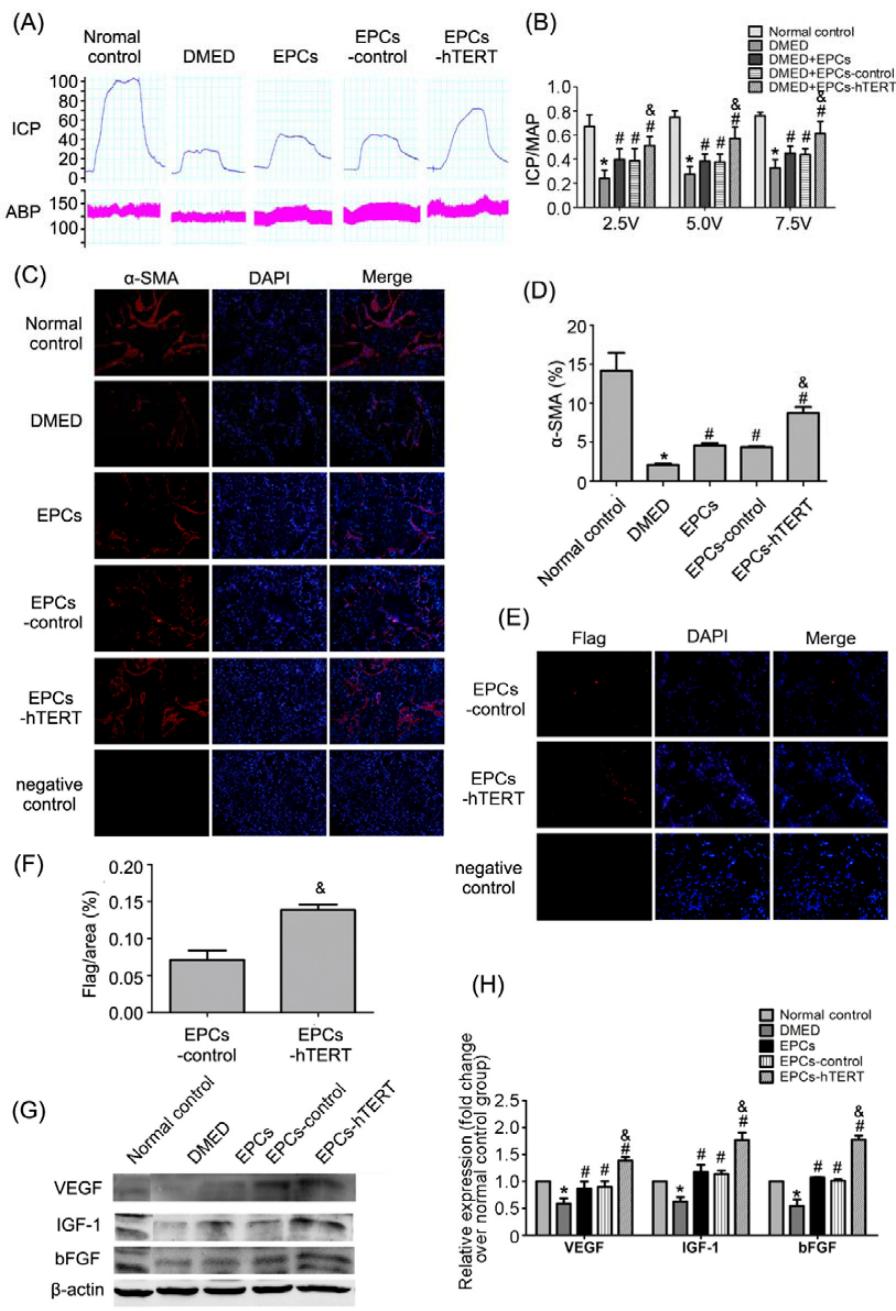
Recovery of erectile function in DMED rats after cell transplantation (A) Representative ICP and MAP tracings in response to cavernous nerve stimulation (5 V, 60 s) in the normal control group, DMED group, EPCs group, EPCs-control group and EPCs-hTERT group. (B) The ratio of ICP/MAP was calculated for each group. (C) Smooth muscle in the corpus cavernosum in each group using immunofluorescence staining (100×). (D) Percentage of smooth muscle in corpus cavernosum shown as relative expression of α-SMA compared with the normal control group. (E) Incorporation of EPCs in the corpus cavernosum after transplantation in EPC-control and EPC-hTERT groups was evaluated using immunofluorescence staining (200×). (F) Results of retained cell quantification expressed as integrated optical density (IOD) of flag–positive area in the corpus cavernosum. (G) Growth factors in penile tissues were detected using Western blotting. (H) Relative quantification of growth factors in penile tissues. *P<0.05 vs. normal control group, # P<0.05 vs. DMED group, & P<0.05 vs. EPCs group and EPCs-control group

The DMED group exhibited a significant decrease in ICP/MAP at all levels of stimulation (2.5, 5.0, and 7.5 volts) compared with the normal control group (all *P*<0.05). Transplantation of EPCs in DMED rats was associated with increased ICP/MAP at all stimulation voltages (all *P*<0.05 *vs*. DMED group). Similarly, the EPCs-control group exhibited increased erectile responses compared with the DMED group (all *P*<0.05). The ICP/MAP of EPCs-hTERT was significantly increased compared with the DMED, EPCs and EPCs-control groups (all *P*<0.05, Figure 4A and 4B). Figure 4C and 4D revealed that smooth muscle content decreased significantly in the DMED group compared with the normal control group (*P*<0.05). Treatment with EPCs or EPCs-control significantly improved the smooth muscle content of corpus cavernosum compared with the DMED group (*P*<0.05). The content of smooth muscle of the corpus cavernosum was increased in the EPCs-hTERT group compared with the EPCs group (*P*<0.05). More flag-positive cells were identified in the corpus cavernosum of the EPCs-hTERT group compared with the EPCs-control group (*P*<0.05) (Figure 4E and 4F). Figure 4G and 4H*P*<0.05). Growth factor levels in the EPCs-hTERT group were significantly increased compared with the EPCs and EPCs-control groups (*P*<0.05).

### Effects of EPCs-hTERT transplantation on cavernous fibrosis

**Figure 5:**
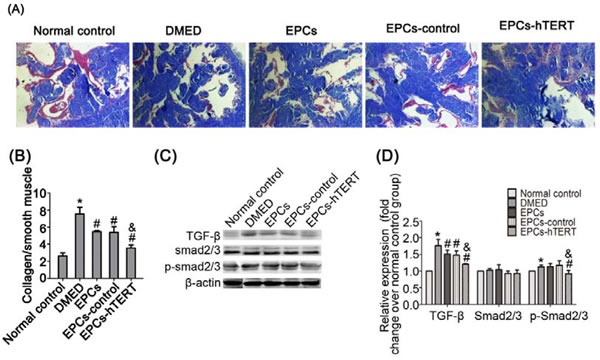
EPCs-hTERT-induced changes in cavernous fibrosis and TGF-β1 signaling pathway expression (A) Masson's trichrome staining: collagen fibers are stained blue, and smooth muscle are stained red (100×). (B) Semi-quantitative evaluation of collagen/smooth muscle ratios. (C) Western blotting was used to detect the expression of TGF-β1, Smad2/3 and p-Smad2/3 in penile tissues of the five groups. (D) Bar graphs show the densitometric ratios of TGF-β1 toβ-actin and phosphorylated Smad2/3 to total Smad2/3. *P<0.05 vs. normal control group, # P<0.05 vs. DMED group, & P<0.05 vs. EPCs group and EPCs-control group.

The collagen/smooth muscle ratio markedly increased in the DMED group compared with the normal control group (*P*<0.05, Figure 5A)). The ratios in the treated groups were decreased compared with the DMED group. The EPCs-hTERT group exhibited a significantly decreased collagen/smooth muscle ratio compared with the EPCs and EPCs-control groups (*P*<0.05, Figure 5B). Western blotting demonstrated that transforming growth factor-β1 (TGF-β1) expression increased markedly in the DMED group. EPCs and EPCs-control groups exhibited a slightly suppressive effect on TGF-β1 expression. Treatment with EPCs-hTERT significantly reduced TGF-β1 expression. Differences in Smad2/3 expression between the five groups were not significant. However, the DMED group exhibited significantly increased Smad2/3 phosphorylation compared with the normal control group (*P*<0.05). The EPCs and EPCs-control groups exhibited slightly decreased Smad2/3 phosphorylation. Treatment with EPCs-hTERT significantly suppressed Smad2/3 phosphorylation (*P*<0.05, Figure 5C and 5D).

### Effects of EPCs-hTERT transplantation on apoptosis in penile tissues

**Figure 6:**
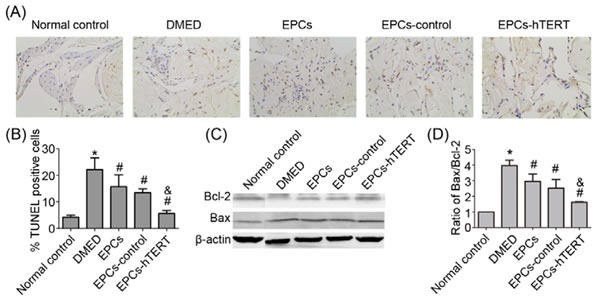
EPCs-hTERT-induced changes in the apoptotic index and expression of apoptosis-related protein levels (A) Apoptotic cells were stained using the TUNEL method (100×). (B) The apoptotic index represents the percentage of apoptotic cells within the total number of cells in a given area. (C) Western blotting was used to evaluate the expression of Bcl-2 and Bax. (D) Bar graph depicts the ratio of Bax and Bcl-2. *P<0.05 vs. normal control group, # P<0.05 vs. DMED group, & P<0.05 vs. EPCs group and EPCs-control group.

Figure 6A and 6B indicate that the number of TUNEL-positive cells in the DMED group was significantly increased compared with the normal control group (*P*<0.05). The number of TUNEL-positive cells was significantly reduced after EPCs or EPCs-control treatment (*P*<0.05). The number of TUNEL-positive cells was decreased in the EPCs-hTERT group compared with the EPCs group. B-cell lymphoma 2 (Bcl-2) and Bcl-2-associated X protein (Bax) expression was measured using western blotting to further study diabetes-induced apoptosis in penile tissues. Figure 6C and 6D indicate that the diabetic state was associated with a significant increase in the Bax/Bcl-2 ratio compared with the normal control group (*P*<0.05). This diabetes-induced increase was significantly suppressed following treatment with EPCs, EPCs-control or EPCs-hTERT (*P*<0.05).

### Effects of EPCs-hTERT transplantation on eNOS and neuronal nitric oxide synthase (nNOS) expression and nitric oxide (NO)-cyclic guanosine monophosphate (cGMP) concentration in penile tissues

**Figure 7:**
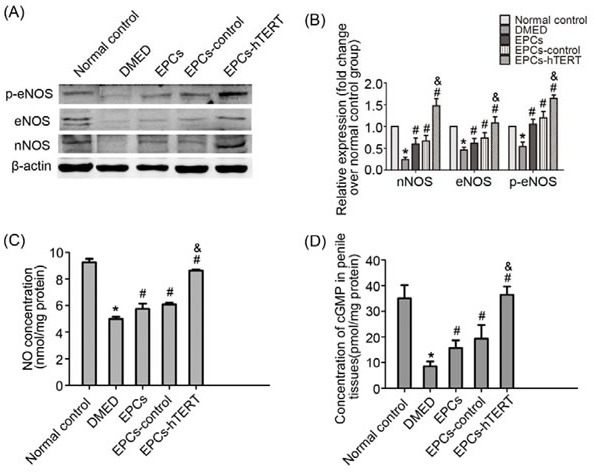
EPCs-hTERT-induced changes in protein expression of eNOS and nNOS and NO-cGMP levels (A) Western blotting was used to detect the expression of eNOS and nNOS. (B) Bar graphs show the relative expression of eNOS and nNOS normalized to β-actin levels. (C) Cavernous NO levels were determined. (D) Cavernous cGMP levels were determined. *P<0.05 vs. normal control group, # P<0.05 vs. DMED group, & P<0.05 vs. EPCs group and EPCs-control group.

Expression of phospho-eNOS (p-eNOS, ser1177), eNOS and nNOS in penile tissues was measured using Western blotting. P-eNOS, eNOS and nNOS protein levels were dramatically decreased in penile tissues of the DMED group compared with the normal control group (*P*<0.05). EPCs or EPCs-control transplantation slightly increased eNOS and nNOS protein levels in diabetic ED rats. The p-eNOS, eNOS and nNOS protein levels in penile tissues of EPCs-hTERT were significantly increased compared with the EPCs group and EPCs-control group (*P*<0.05, Figure 7A and 7B). Concentrations of NO and cGMP were detected to further explore the NO-cGMP signaling pathway. Figure 7C and 7D indicate that NO and cGMP penile concentrations in the DMED group were significantly attenuated compared with the normal control group (*P*<0.05). The EPCs group and EPCs-control group exhibited slight increases in NO and cGMP concentrations, and the EPCs-hTERT group exhibited significantly increased NO and cGMP levels compared with the DMED group (*P*<0.05).

## DISCUSSION

Various reports demonstrated a therapeutic effect of stem cells in animal studies of ED [19, 22, 23]. This study demonstrated that the overexpression of hTERT significantly enhanced the therapeutic effect of EPCs in the restoration of electrically induced erectile function in DMED rats.

Increasing evidence suggests that TERT plays an important role in mediating cell proliferation and antiapoptotic functions against various cytotoxic stresses independent of telomerase activity [17, 24–26]. Our study demonstrates that EPCs-hTERT exhibited increased proliferative capacity. An increased number of cells in S phase and a reduced number of cells in the G1 phase of the cell cycle may contribute to the enhanced cellular proliferation. This effect is associated with Cyclin D1, cell division cycle 6 (CDC6) expression, and the hyperphosphorylation of retinoblastoma tumor suppressor protein (RB) at the molecular level [27]. TERT overexpression enhances the expression of growth-promoting genes and suppresses the expression of growth-inhibitory genes [28]. The effect of TERT on proliferation was also demonstrated in vivo. Upregulation of TERT expression in K5-mTert mice promotes epidermal stem cell proliferation and hair growth in the absence of changes in telomere length [29].

TERT overexpression induces resistance to oxidative stress and other genotoxic insults[30]. EPCs-hTERT cells were more resistant to oxidative-stress-induced cell death. The mechanism of the protective effect depends on the catalytic activity of TERT, which antagonizes the p53 pathway and binds to mitochondrial DNA [17, 30]. Moreover, TERT is excluded from the nucleus under increased oxidative stress in a dose- and time-dependent manner. Extranuclear TERT improved mitochondrial function and decreased retrograde response [17]. We found that hTERT protein was primarily expressed in the cytoplasm of EPCs-hTERT. The intracellular peroxide concentration is significantly reduced compared with wild-type cells [27], which implies a greater resistance to oxidative stress. Similarly, ROS levels in EPCs-hTERT were significantly reduced compared with EPCs and EPCs-control.

The multipotency of stem cells was proposed as the potential mechanism for the therapeutic effect on restoring erectile function in rats. Stem cells transplanted into rat corpus cavernous have the potential to differentiate toward endothelial cells or smooth cells [31]. However, very few stem cells were successfully tracked after intracavernous application in a series of studies for ED treatments using stem cells [18, 32]. Oxidative stress and other irregular microenvironments may contribute to the loss of transplanted stem cells. Therefore, we should identify novel methods to retain more stem cells in penile tissues. Our study demonstrated that more EPCs-hTERT cells were retained in penile tissues. The enhanced proliferation and anti-oxidant capacity may contribute to the retention of stem cells. However, the amount of EPCs-hTERT retained in corpus cavernous was still too small to explain the therapeutic effect, which was similar to previous studies [18, 32] and indicates that other mechanisms may exist.

The secretion of trophic growth factors emerged as the main mechanism to explain the therapeutic effect of stem cells in the treatment of ED [33, 34]. Various studies demonstrated that stem cells secrete many growth factors, including CXCL5, adrenomedullin, bFGF, VEGF, nerve growth factor and BDNF [19, 34–36]. These growth factors exhibit potent angiogenic and neurotrophic activities. Our study found that EPCs secreted VEGF, IGF-1 and bFGF, which may contribute to the decreased corporal apoptosis and fibrosis and increased NO-cGMP signaling pathway. We demonstrated for the first time that the amount of trophic growth factors secreted by EPCs-hTERT was dramatically increased compared with EPCs and EPCs-control *in vitro*. The enhanced paracrine character and greater number of retained cells in penile tissues may contribute to the increased amount of growth factors *in vivo*. Adipose-derived stem cells (ADSCs), which are immortalized by hTERT combined with SV-40 or HPV E6/E7, demonstrated an elevated capability to secrete hepatocyte growth factor (HGF) and VEGF [37]. However, the mechanism for this effect is unclear, but it may be associated with hTERT-mediated promotion of the expression of trophic factors [38].

There are certain limitations in this work. EPCs secreted many types of growth factors. Our study only selected VEGF, IGF-1 and bFGF because these three growth factors are the most commonly studied for the treatment of ED[39]. The relationship of the concentration of these growth factors to the therapeutic effect of EPCs-hTERT treatment on DMED and the underlying mechanisms are not clarified. Further studies are needed to understand the exact mechanism.

## CONCLUSIONS

This study demonstrated that hTERT enhanced proliferation, anti-oxidant capacity and the paracrine character of EPCs. Intracavernous injection of EPCs-hTERT more efficiently decreased corporal apoptosis and fibrosis but increased eNOS and nNOS expression as well as NO and cGMP concentrations to promote the recovery of DMED. The potential mechanisms of the recovery are likely attributed to the greater retention of cells in penile tissues and the paracrine effects of EPCs-hTERT.

## MATERIALS AND METHODS

### Isolation and culture of EPCs

EPCs were isolated from rat bone marrow as previously described [13]. Briefly, bone marrow was harvested and density gradient centrifugation with Percoll (Sigma-Aldrich, St. Louis, USA) was performed to isolate bone marrow-derived mononuclear cells (BM-MNCs). Isolated cells were resuspended in EGM-2 (Lonza, Walkersville, USA) containing 2% fetal bovine serum (FBS) plus growth factors, including bFGF, epidermal growth factor (EGF), VEGF and IGF-1. Cells were cultured in flasks at 37°C with 5% CO_2_, and non-adherent cells were removed after overnight culture. The medium was exchanged every 3 days.

### Cell differentiation studies

#### Adipocyte differentiation

EPCs were seeded at a density of 1×10^4^ cells/cm^2^ in 6-well plates and cultured in EGM-2 until the cells were confluent. The medium was replaced with low-glucose Dulbecco's Modified Eagle's medium (DMEM) containing 10% FBS, 1 mmol/L dexamethasone, 0.5 mmol/L isobutyl methyl xanthine (IBMX), 50 mg/L indomethacin, and 10 mg/L insulin (all from Sigma-Aldrich). This adipogenic medium was changed every 2 days. Cells were differentiated for 21 days, washed twice with PBS and fixed with 4% paraformaldehyde for 15 minutes. Lipid staining was performed with a filtered 0.3% Oil red-O staining solution.

#### Smooth muscle cell differentiation

EPCs were cultured at 0.5×10^4^ cells/cm^2^ in 12-well plates and grown for 24 hours in EGM-2. The medium was replaced by low-glucose DMEM containing 10% FBS, 5 μg/L TGF-β1 and 50 μg/mL platelet-derived growth factor-BB (PBGF-BB) (all from PeproTech, Rocky Hill, United States). The medium was refreshed every 2 days.

### Immunofluorescence and histological analysis

Cells were fixed with 4% paraformaldehyde for 20 minutes and permeabilized with 1% Triton X-100 in PBS for 30 minutes followed by blocking with 1% bovine serum albumin (BSA) for 1 hour. The cells were incubated for 24 hours with primary antibodies. Antibodies against CD31 (1:100, Boster, Wuhan, China), CD34 (1:100, Boster), CD133 (1:100, ProteinTech, Chicago, USA), VEGF receptor-2 (VEGFR-2, 1:100, Affinity, Zhenjiang, China) were used to verify EPCs phenotype. TERT (1:200, Affinity) and flag (1:200, Affinity) was used to demonstrate the distribution of TERT protein in EPCs-hTERT. α-smooth muscle actin (α-SMA, 1:100, Boster) was used to identify EPCs that were induced into smooth muscle cells. Cells were incubated for 24 hours, washed extensively with PBS and incubated with the appropriate secondary antibodies for 1 hour. Nuclei were stained with diamidino-2-phenyl-indole (DAPI).

Rat penile sections were dewaxed in dimethylbenzene and washed thrice with PBS. The slides were blocked and incubated with the following primary antibodies. α-SMA (1:100, Boster) was used to evaluate smooth muscle content in penile tissues, and flag (1:100, Affinity) was used to detect EPCs retained in penile tissues. Slides were incubated with the appropriate secondary antibodies. Masson's trichrome staining was performed to determine the ratio of cells to collagen in penile tissues of all groups. Digital images were obtained using an Olympus DP71 fluorescence microscope.

### Western blotting

Penile tissues or cultured cells were extracted in ice-cold lysis buffer. Protein (50 μg) were loaded on 12% sodium dodecyl sulfate/polyacrylamide (SDS-PAGE) gels and transferred to polyvinylidene difluoride membranes. Membranes were blocked with 5% BSA for 2 hours and incubated with the following primary antibodies: TERT (1:1000, Affinity), VEGF (1:1000, ProteinTech), bFGF (1:1000, Affinity), IGF-1 (1:1000, ProteinTech), TGF-β1 (1:1000, Abcam, Cambridge, UK), Smad2/3 (1:1000, Abcam), phospho-Smad2/3 (p-Smad2/3, 1:1000, Abcam), Bcl-2 (1:1000, Affinity), Bax (1:1000, ProteinTech), eNOS (1:1000, Abcam), p-eNOS (1:1000, CST, Danvers, USA), and nNOS (1:1000, Abcam). Membranes were washed and incubated with the appropriate secondary antibodies (1:5000) for 1 hour at room temperature. Protein bands were detected using an enhanced chemiluminescence detection system (Pierce; Thermo Fisher Scientific, Rockford, USA).

### Tube formation assay of rat EPCs

A matrigel (BD, San Jose, USA) was thawed at 4°C overnight and used to pre-coat a 96-well plate. 1×10^4^ EPCs were placed on the top of the matrigel. After 8 h of incubation at 37°C, networks of tubes were photographed using a microscope.

### hTERT transduction of rat EPCs

Third-passage EPCs were cultured in a 6-well plate at a density of 2×10^6^ cells for 24 hours. Lentivirus with *hTERT* and flag tags infected EPCs at a multiple of infection (MOI) of 50. The medium was removed after 24 hours, and the EPCs were recultured in normal medium for 48 hours. Infected cells were selected using puromycin (5 μg/mL) in EGM-2. Surviving cells were noted as first passage EPCs-hTERT.

### Real-time quantitative PCR analysis

Total RNA was extracted using a multisource RNA miniprep kit (Corning, NY, USA) converted to cDNA using the PrimeScript^TM^ RT reagent kit (TaKaRa, Dalian, China). Real-time quantitative polymerase chain reaction (PCR) was performed using SYBR Premix Ex Taq (TaKaRa) on an MX3000P quantitative PCR system (Agilent, Santa Clara, USA). The following cycling conditions were used: pre-denaturation at 95°C for 30 seconds; 40 cycles at 95°C for 5 seconds, 60°C for 30 seconds and 72°C for 30 seconds. The level of β-actin mRNA expression was measured as an endogenous reference and used for normalization purposes. Relative quantification of the expression levels of each transcript for each group was calculated using the 2^−ΔΔCt^ method. We used the following PCR primers: hTERT_s: 5′-GACGGTGTGCACCAACACATCTA-3, hTERT_a: 5′-TTCTTGGCTTTCAGGATGGAG-3′, rat TERT (rTERT)_s: 5′-GCTGGACACTCGGACTTTGGA-3′, rTERT_a: 5′-ACTTCAACCGCAAGACTGACAAGA-3′, β-actin_s: 5′-GACGGTGTGCACCAACATCTA-3′, β-actin_a: 5′-TTCTTGGCTTTCAGGATGGAG-3′.

### Measurement of telomerase activity

Telomerase activity was evaluated using a telomeric repeat amplification protocol (TRAP) telomerase detection kit (Zhongxiyuanda, Beijing, China). The procedure was performed according to the manufacturer's instructions. Cells were collected and lysed with CHAPS buffer. Proteins extracted from cells were used for reaction. Polyacrylamide gel electrophoresis was performed, and gels were stained using the silver nitrate method.

### Cell proliferation assay

Cells were plated in 96-well plates at a density of 2×10^4^/mL and cultured overnight. Cells were incubated with 5-ethynyl-2′-deoxyuridine (EdU) for 4 hours before fluorescent detection. Cells were fixed with 4% paraformaldehyde for 15 minutes at room temperature and stained with a Cell-Light™ EdU Apollo®488 In Vitro Imaging Kit (RioBio, Guangzhou, China) in accordance with the manufacturer's instructions.

### EPCs-hTERT survival in oxidative stress

Cells were seeded in 96-well plates at density of 1×10^4^ cells/cm^2^ overnight and cultured in EGM-2 supplemented with 400 μmol/L hydrogen peroxide for 24 hours. The CCK-8 assay was used to detect cell survival. Cells were incubated with 10 μL CCK-8 (Dojindo, Shanghai, China) for 2 hours, and cell density was determined using a multiscan MK3 microplate reader (Thermo Fisher Scientific, Waltham, USA) at 450 nm. All experiments were performed in triplicate and repeated independently thrice.

### Measurement of intracellular ROS

Intracellular ROS was detected using an ROS assay kit (Beyotime, Nantong, China) according to the manufacturer's instructions. Cells at passage 10 were seeded in a 96-well plate at a density of 2×10^4^ cells/cm^2^ overnight and treated with dichloro-dihydro-fluorescein diacetate (DCFH-DA, 10 μmol/L) for 30 minutes. Cells were washed with PBS and imaged using a fluorescent microscope.

### Paracrine characteristics

EPCs, EPCs-control, and EPCs-hTERT were seeded at a density of 2×10^6^ cells per 175-cm^2^ flask and cultured for 24 hours. Medium was exchanged for serum-free EGM-2 without growth factors and incubated for 24 hours. Cell supernatants were collected and concentrated to 100 μL using Amicon® Ultra-4 Centrifugal Filter Units (Merck Millipore, Darmstadt, Germany). Western blotting was used to evaluate paracrine characteristics.

### Establishment of DMED rat models

Animal studies were performed in accordance with relevant guidelines and regulations. The Tongji Hospital, Tongji Medical College, Huazhong University of Science and Technology Animal Care and Use Committee approved the protocols. Forty 8-week-old male Sprague-Dawley (SD) rats were intraperitoneally injected with streptozotocin (STZ, 60 mg/kg, Sigma-Aldrich)[45]. Rats with blood glucose concentrations greater than 16.7 mmol/L 3 days after STZ injection were considered diabetes. Diabetic rats were selected 8 weeks later using the apomorphine (APO) test[46]. Briefly, each rat received a subcutaneous injection of APO (80 mg/kg, Sigma-Aldrich) in the loose skin of neck. Erectile responses, including back arching, pelvic thrusting followed by integral movements of the engorged glans penis and shaft, and immediate grooming of the genital area, were recorded for 30 minutes. Rats that did not exhibit an erectile response were considered DMED rats.

### Transplantation of EPCs to DMED rat corpus cavernous

Twenty-four DMED rats were randomly divided into four groups: DMED rats without treatment (DMED group), DMED rats treated with EPCs (EPCs group), DMED rats treated with EPCs-control (EPCs-control group), and DMED rats treated with EPCs-hTERT (EPCs-hTERT group). The normal control group included six normal rats. DMED rats were anesthetized via intraperitoneal injections of sodium pentobarbital (40 mg/kg) and placed in a supine position. The penis was exposed, and an elastic band was placed at the base of the penis. A total of 5×10^5^ cells was dissolved in 70 μL PBS and injected into the corpus cavernous. The elastic band was removed 3 minutes after the injection.

### *In vivo* evaluation of erectile function

Erectile function was evaluated 2 weeks after cell transplantation using electric stimulation as previously described[45]. Briefly, rats were anesthetized using sodium pentobarbital (40 mg/kg). A PE-50 tube was cannulated into the left carotid artery and connected to a data acquisition system (AD Instruments Powerlab/4SP, NSW, Australia) to continuously record arterial blood pressure (ABP). The cavernous nerves were exposed at the posterolateral aspect of the prostate on both sides. A 25-gauge needle was inserted into the right crus to continuously monitor ICP. Stimulation of the cavernous nerve was performed through a bipolar electrode at 15 Hz frequency for 1 minute, and the erectile response was measured at different voltages (2.5, 5.0 and 7.5 volts) with a 3-minute interval between stimulations. The ratio of maximal ICP and MAP was calculated to evaluate erectile function.

### TUNEL assay

Penile sections were stained using an In Situ Cell Death Detection kit (Roche Applied Science, Indianapolis, USA). Briefly, sections were deparaffinized, rehydrated, and treated with nuclease-free proteinase K (20 mg/L) for 20 minutes. Sections were washed in PBS and incubated with the TUNEL reaction mixture containing terminal deoxynucleotidyl transferase and dUTP for 1 hour in a humidified chamber at 37°C. After washing in PBS, sections were incubated in converter peroxidase-conjugated anti-fluorescein (POD) antibodies and stained with diaminobenzidine (DAB) solution.

### cGMP measurement

The cGMP concentration measurement was perform with an enzyme-linked immunosorbent assay kit (Westang, Shanghai, China) according to the manufacture's instructions.

### NO measurement

The NO concentration was measured using total nitric oxide assay kit (Beyotime) according to the manufacture's instructions.

### Statistical analysis

Data are presented as the means ± standard deviation (SD). Statistical significance was assessed using Student's *t*-test for comparisons of two groups or one-way analysis of variance followed by a *post hoc* (Tukey) test for comparisons between multiple groups. Intergroup differences were considered statistically significant at a *P*-value<0.05.

## SUPPLEMENTARY MATERIAL FIGURE


