# Supplementary material for: Treatment of diabetes mellitus-induced erectile dysfunction using endothelial progenitor cells genetically modified with human telomerase reverse transcriptase
Source: Oncotarget. 2016 Jun 7;7(26):39302–15. doi: 10.18632/oncotarget.9909 (PMC5129934; doi:10.18632/oncotarget.9909)
Supplement: Supplementary file 1 [file oncotarget-07-39302-s001.pdf]

Treatment of diabetes mellitus-induced erectile dysfunction using endothelial progenitor cells genetically modified with human telomerase reverse transcriptase

Supplementary Material

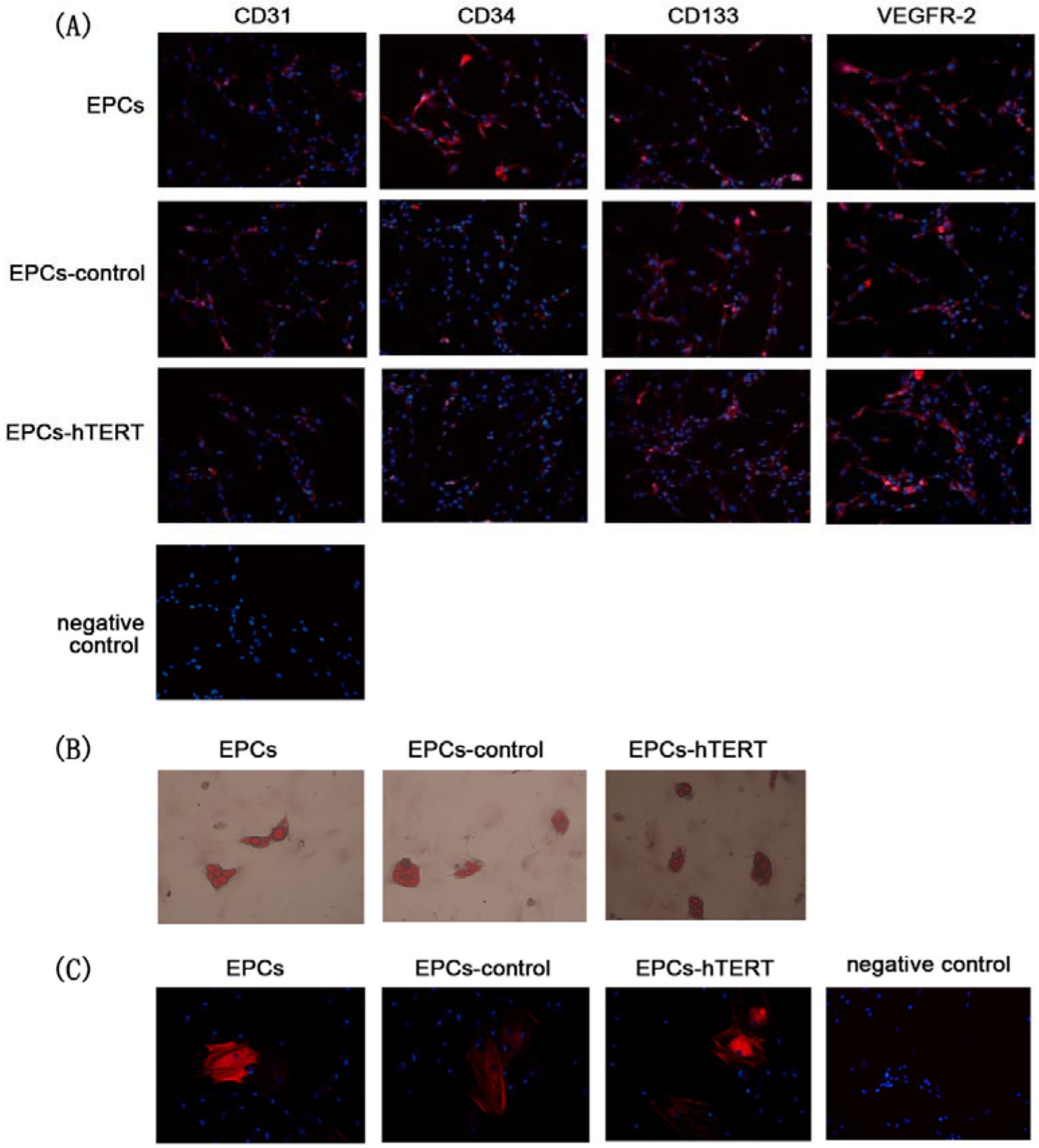

Supplemental figure 1. Phenotypic profile and differentiation potential of EPCs-hTERT. (A) CD31, CD34, CD133 and VEGFR-2 expression was detected using immunofluorescence (200×). (B) Adipogenesis of EPCs-hTERT was demonstrated using oil red-O staining (200×). (C) EPC-hTERT differentiation potential into smooth muscle cells was demonstrated using immunofluorescence staining (200×).
